# Oxidative modification and aggregation of creatine kinase from aged mouse skeletal muscle

**DOI:** 10.18632/aging.100055

**Published:** 2009-05-22

**Authors:** Jonathan E. Nuss, James K. Amaning, C. Eric Bailey, James H. DeFord, Vincent L. Dimayuga, Jeffrey P. Rabek, John Papaconstantinou

**Affiliations:** Department of Biochemistry and Molecular Biology, University of Texas Medical Branch, Galveston, TX 77555-0643, USA

**Keywords:** Creatine kinase, creatine kinase structure, creatine kinase kinetics, 3-nitrotyrosine, carbonylation, skeletal muscle, aging

## Abstract

Creatine kinase catalyzes the reversible transfer of the gamma phosphate from ATP to creatine forming the high energy compound creatine phosphate.  Muscle creatine kinase (CKm) activity maintains energetic homeostasis as variations in energy requirements dictate that ATP be readily available.  Recent studies suggest that CKm activity is altered during aging.  Proteomic analyses have shown that CKm is 3-nitrotyrosine (3-NT) modified and carbonylated in aged rodent skeletal muscle.  However, it remains unknown if these modifications affect its structure and activity. To address this we characterized oxidatively modified CKm from the quadriceps of young, middle-aged, and aged mice. Our data indicate that 3-NT modified and carbonylated CKm are found predominantly in aged muscle and that it exists in high molecular weight oligomers and insoluble protein aggregates. CKm from middle-aged and aged mouse quadriceps also exhibits structural instability that may account for its reduction in function. These structural and functional changes correlate with the differential protein modifications. Interestingly, the majority of the age-related changes in enzyme activity and protein stability occurred by middle age. Our studies indicate that the age-associated oxidative and nitrative modification of CKm results in a decrease in its activity and may cause structural changes that promote oligomerization and aggregation.

## Introduction

Creatine kinase (CK) is an essential enzyme found in tissues with periodic fluctuations in energetic requirements, such as skeletal muscle, cardiac muscle and the brain [1]. CK catalyzes the reversible transfer of the gamma phosphate from ATP to creatine forming creatine phosphate (CrP) and ADP. The cycling of creatine and CrP play an important homeostatic role as CK catalyzes the synthesis of ATP from CrP and ADP when energy requirements are high, such as during exercise. During periods of rest, creatine phosphate pools are replenished as CK catalyzes the reverse reaction [1]. Within skeletal muscle cytosol, the majority of CK activity is attributed to the homodimeric muscle isozyme of CK (CKm); the brain isozyme, (CKb) is also found in muscle cytosol, though at significantly lower concentrations [2].

Muscle type CK has the unique property of binding with the M-line of sarcomere [3]. Its catalytic activity, which involves its function in muscle is elaborately regulated.  In its activated form acidification of the microenvironment stimulates its binding with M-line proteins [4] where it supplies ATP coupled with myofibrillar actin-activated Mg^2+^-ATPase [5,6].  In the resting state it dissociates from the myofibril and catalyzes the formation of phosphocreatine to reserve energy [4].  Recent studies have shown that a negative regulation of CKm occurs through its oxidation O-CKm which is then targeted for degradation via the ATP-ubiquitin-proteome system in muscle cells.  This oxidation occurs via the formation of an intrachain disulfide bond between Cys^74^ and Cys^146^ [7].  Interestingly, circular dichroism (CD) analysis, intrinsic fluorescence and ANS fluorescence have shown that O-CK has decreased secondary structure, including increased hydrophobic surface exposure.  Functionally, the O-CKm showed a significant decrease in enzyme activity and the loss of ability to interact with the M-line protein, myomesin [7].

Reduction of CKm activity may be a major contributor to the gradual loss of muscle function associated with aging.  Several lines of investigation have shown age-related reductions in skeletal muscle oxidative capacity in rodents and humans [8,9]. Additionally, recent proteomic-liquid chromatography-tandem mass spectrometry (LC-MS/MS) experiments have definitively shown that CKm is 3-nitrotyrosine (3-NT) modified within aged skeletal muscle and a novel approach using the fluorescent probe 4,4-dianilino-1,1-binaphthyl-5,5-disulfonic acid (BisANS) suggests that the three dimensional structure of CKm is altered during aging [10-12]. Furthermore, in crude extracts prepared from human brains, reduced activities for aged samples compared to young controls parallel the increases of CKb carbonylation [13].  However, the consequences of oxidative modification of CKm to its structure and function and its contribution to the age-related decrease in skeletal muscle function is not understood.

Though a growing body of literature suggests that CKm activity might be altered during aging, a detailed structure and function analysis of oxidatively modifiedCKm isolated from animals of different ages has not been performed. These experiments are essential to demonstrate that the structure and function of oxidatively modified CKm are alteredin aging skeletal muscle. To address these issues we purified and characterized CKm from the quadriceps of young, middle-aged and aged mice. Circular dichroism, limited proteolysis, and enzyme kinetic analysis demonstrated reduced stability and enzyme activity for CKm obtained from middle-aged and aged mice relative to young mice.  Interestingly, our fractionation of purified CKm revealed a chromatographic shift of tyrosine nitrated CKm vs. unmodified as well as carbonylated enzyme.  Finally, as with the brain studies [13] the age-associated reductions in function and stability correlated with levels of protein nitration and carbonylation. In addition, the procedure of purification of 3-NT modified CKm resulted in the identification of an apparent trimeric form of CKm, suggesting that 3-NT modifycation may lead to the oligomerization and aggregation of this enzyme.

Our results indicate that there is an age-associated increase in nitrative modification and carbonylation to CKm, that these modifications correlate with significant decreases in activity and that these modifications may induce structural changes that promote oligomerization and aggregation. Overall, these data support a model of skeletal muscle aging where reduction of CKm activity may be due to oxidative modifications that may contribute to diminished muscle function.

## Results

### Purification of CKm from young, middle-aged, and aged mouse quadriceps

**Figure 1.**
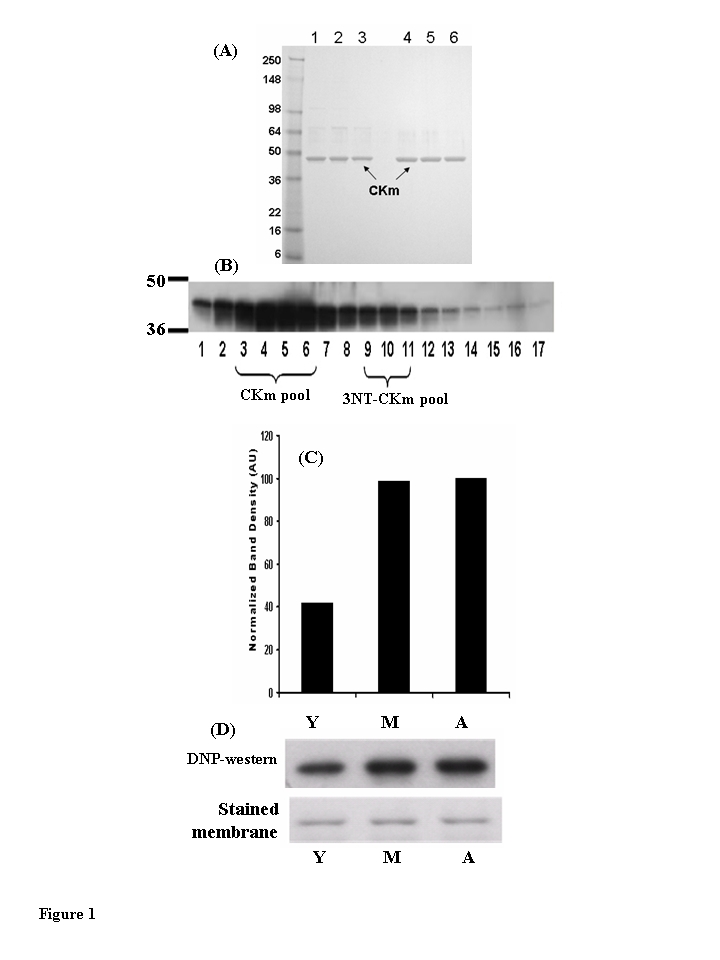
Muscle creatine kinase (CKm) purified from young (3-6 months), middle aged (12-14 months) and aged (20-24 months) mouse quadriceps. (**A**) Peak Blue Sepharose fractions of purified CKm (1 μg) from young (lane 1), middle aged (lane 2) and aged (lane 3) mouse muscle were resolved on a denaturing SDS gel and stained with Coomassie Blue.  These fractions are ~85% purified CKm and were used for enzyme kinetic analyses.  CKm within side fractions from the Blue Sepharose pH gradient elution were pooled and purified to a single band using hydroxyapatite chromatography.  Lanes 4-6 represent samples from young, middle-aged and aged mice, respectively.  (**B**) Western blot analysis of CKm levels in fractions eluted from a Blue Sepharose column, using an anti-creatine kinase type M antibody.  (**C**) Densitometric analysis demonstrates the increase in carbonylated CKm in quadriceps of young, middle aged and aged mice.  The peak level of carbonylation occurs in muscle of middle aged mice.  (**D**) immunoblot analysis of carbonylated CKm in Blue Sepharose fractions [3-6],  The carbonylated CKm was identified by anti-DNP antibody.

To directly examine age-related changes in protein structure and function, CKm was purified from the quadriceps of young, middle-aged, and aged mice (Figure 1A). An affinity Blue Sepharose chromatography procedure using a sequential isocratic pH elution followed by a gradient pH elution, resulted in CKm that was greater than 85% pure (Figure 1A, lanes 1-3). These samples, from all three age groups, were used in the analysis of CKm enzyme activity and for immunoblotting experiments that compared relative levels of 3-NT and carbonylation modification. CKm protein that was greater than 95% pure (Figure 1A, lanes 4-6) was obtained from all three age groups using an additional hydroxyapatite chromatography step (see Figure 5); these samples were used in CD and limited proteolytic digestion studies. Details of CKm purification are given in Methods.

### Skeletal muscle creatine kinase is 3-nitrotyrosine modified during aging

**Figure 2.**
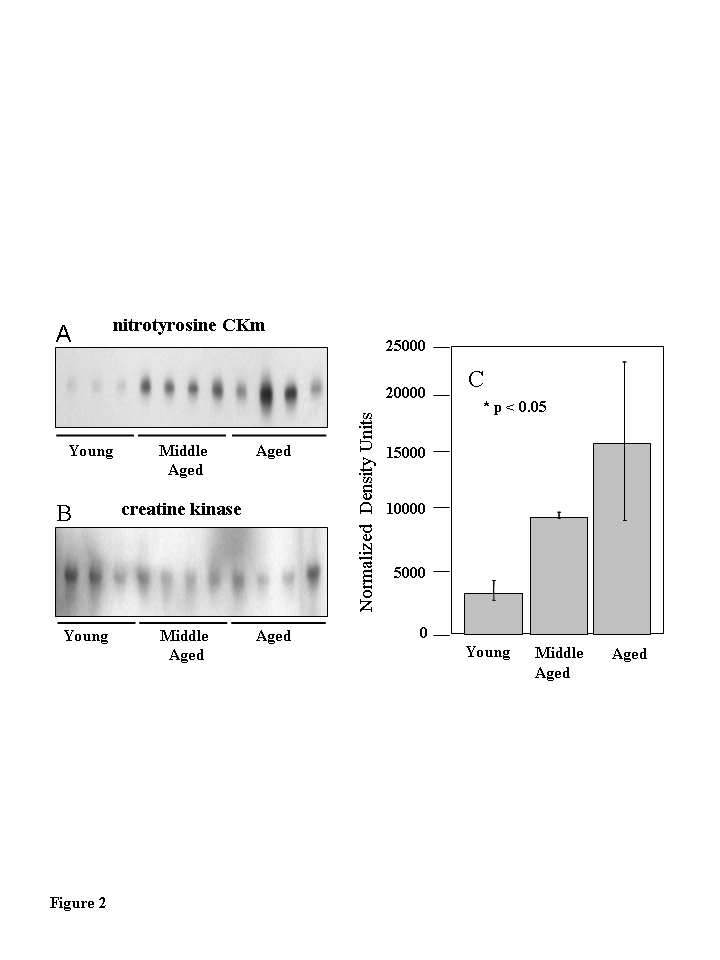
Muscle creatine kinase (CKm) is differentially 3-nitrotyrosine modified during aging. Quadriceps muscle extracts (30 ?g) from six young (3-6 months), six middle-aged (12-14 months) and five aged (20-24 months) old mice were resolved by SDS PAGE and transferred to a PVDF membrane. (**A**) Immunoblots probed with a monoclonal anti-3-nitrotyrosine antibody reveal a nitrated 45 kDa protein in middle aged and aged samples. (**B**) The immunoblots in (**A**) were reprobed with an anti-creatine kinase type M antibody which identifies the levels of CKm in the samples applied to the gels in (**A**). (**C**) Densitometric analysis demonstrates the progressive increase in nitration in the aging muscle samples.  The highest level of nitration is seen in the aged muscle samples relative to the young samples. Error bars depicted on the figure represent calculated standard errors of mean.  p < 0.05 in both cases.

Western blot analysis using a monoclonal anti-3-nitrotyrosine antibody was used to compare levels of 3-NT modification within whole quadriceps extracts obtained from six young (3-6 months), six middle-aged (12-14 months), and five aged (20-24 months) mice (Figure 2A). A band with an apparent molecular weight of ~ 45 kD exhibited a progressively increasing level ofnitration from middle aged to aged samples compared to young samples. Densitometric analysis of the 45 kDa bands shows significantly greater levels of 3-NT immunoreactivitywithin the aged samples (Figure 2A; p<0.05). The anti-nitrotyrosine blot was re-probed with an anti-CKm antibody (Figure 2B).  CKm blots were superimposable with the 3-NT modified 45 kDa band, indicating that the modified protein is CKm.  The protein  identity was confirmed by 2-D gel electro-phoresis and mass spectrometry.  Kanski et al. [10,11] have shown that CKm is 3-NT modified within aged rat skeletal and cardiac muscle. Our studies demonstrate higher levels of 3-NT modifications to CKm in aged mouse muscle, relative to young and middle-aged samples (Figure 2C).

### Purified CKm is carbonylated in an age-dependent manner

The accumulation of oxidized proteins is a characteristic of the aged phenotype and these age-related oxidative modifications have been shown to affect the biological activity of the modified proteins [14-17]. Western blot analysis, using 2,4-dinitrophenyl-hydrazine (DNP) to compare levels of carbonylation within the Blue Sepharose purified CKm samples revealed that the carbonylated protein is within protein fractions 3-6 (Figure 1B; 18, 19).  Densitometric analysis of the blot shows that purified CKm protein from middle-aged and aged mice contains approxi-mately 2.5 times more carbonyl modifications relative to CKm purified from young mice (Figure 1C,D). The observed higher levels of carbonylation in the middle-aged and aged samples correlate with the observed age-associated decreases in CKm activity and stability (see Figure 7).

### Purified CKm and glycogen phosphorylase are nitrated in an age-dependent manner

**Figure 3.**
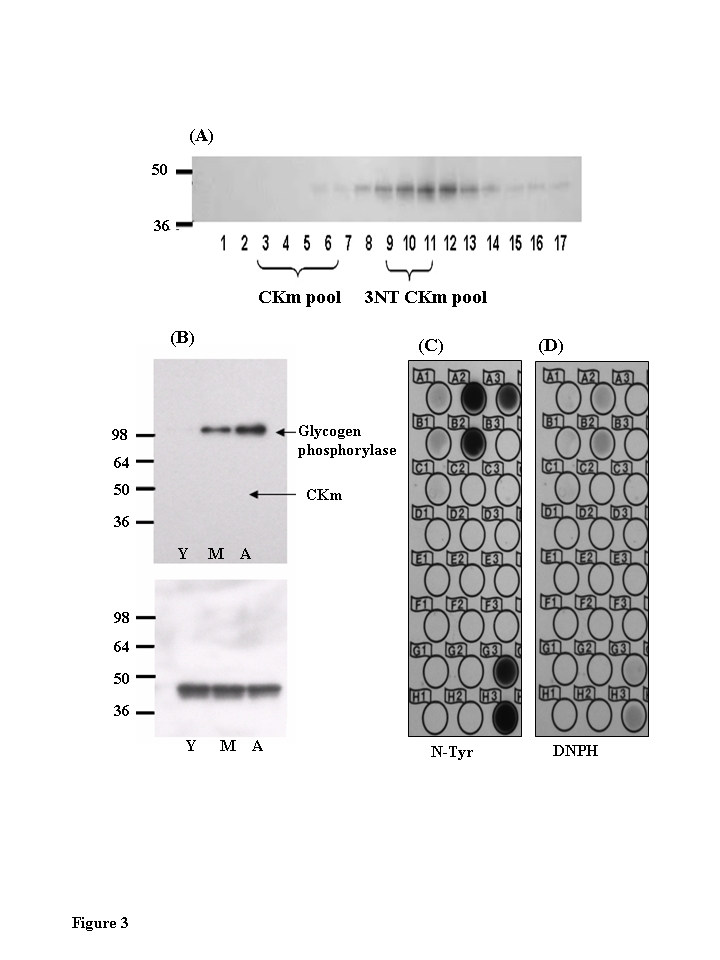
Chromatographic elution properties of 3-nitrotyrosine modified muscle creatine kinase are altered. (**A**) Immunoblots of Blue Sepharose fractions of CKm show that fractions 9-12 are tyrosine nitrated.  CKm (1 μg, > 85 % pure) was resolved on a denaturing SDS polyacrylamide gel, transferred to a PVDF membrane, and analyzed by immunoblot using anti-3NT-antibody. (**B**) Immunoblots of Blue Sepharose fractions 3-7 shows that 3-NT CKm is not detected in these fractions.   Blots probed with an anti-3-nitrotyrosine antibody (top panel) detected nitrated glycogen phosphorylase in middle aged and aged muscle. However, the purified CKm in this fraction is not nitrated. Blots were reprobed with an anti-CK antibody (3B, lower panel). (**C**) Fractions 9-11 were further fractionated by reverse phase chromatography.  These fractions were spotted on to PVDF membranes and analyzed for nitrated CKm using anti-nitrotyrosine antibody, and carbonylated CKm using anti-DNPH antibody (**D**).

CKm within solubilized muscle extract is 3-NT modified (Figure 2). Western blot analysis was used to probe for 3-NT modification within the Blue Sepharose purified CKm samples (Figure 3A). Analysis of fractions 3-6 (Figure 3B) revealed a single band with an approximate molecular weight of 100 kDa that is 3-NT modified in an age-dependent manner.  The highest levels of modification of this protein occurs in the aged muscle.  In a parallel experiment, the band identified as a 3-NT modified protein was excised from a Coomassie Blue stained SDS-containing acrylamide gel.  MALDI-TOF/TOF mass spectrometry analysis identified the modified protein as glycogen phosphorylase (Figure 3B; Table 1). The 3-NT modification of this protein within aged rodent skeletal muscle has been previously observed [11]. The anti-3-NT immunoblot of Fractions 3-6 was reprobed with an anti-CKm antibody (Figure 3B, lower panel); these data show that the same Blue Sepharose purified CKm samples (fractions 3-6) are not 3-NT modified.  On the other hand, immunoblot analysis revealed that fractions 9-12 contain 3-NT modified CKm (Figure 3A).  These data suggest that the nitrotyrosine modification may cause a significant shift in the elution properties of 3-NT modified CKm.  The data also raise the question of whether the nitrated CKm is also carbonylated.  To address this we placed the pooled Blue Sepharose fractions 9-11 on a reverse phase column to determine the levels of nitration vs. carbonylation.  The anti-nitrotyrosine and anti-DNP immunoblots in Figure 3C and 3D respectively clearly show a strong response to the anti-nitrotyrosine whereas the response to anti-DNP is negligible.  These results suggest that the 3-NT modified CKm may not be modified by carbo-nylation and that the elution of the nitrated form is shifted away from the elution of the carbonylated CKm.

**Table 1.** MALDI TOF/TOF Identification of 3-nitrotyrosine modified proteins.

Protein		Mol. Mass (theor./expt.)	Peptide Count	Mascot Protein Scorea	Expectation Valueb
Muscle glycogen phosphorylase [mus musculus]	6755256	97.2/100	42	589	1.2 x 10^-54^
Creatine kinase, muscle [mus musculus]	6671762	43.0/130	16	597	1.9 x 10^-55^

### The level of alpha helical content is reduced in CKm from middle-aged and aged mice

**Figure 4.**
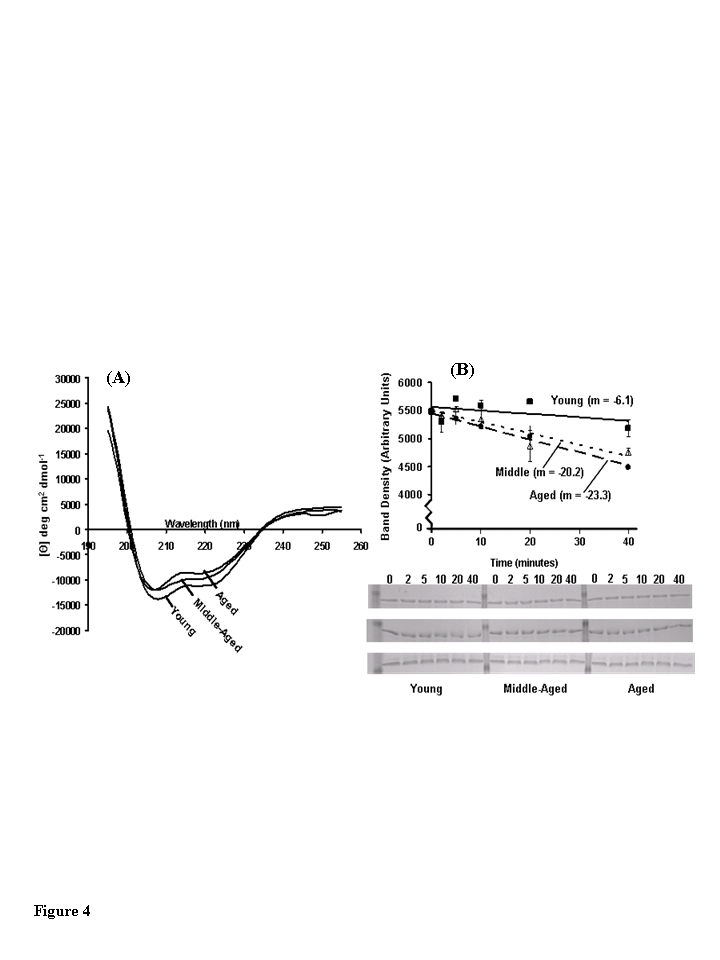
Structural analysis of muscle creatine kinase (CKm) purified from differently aged mouse quadriceps. (**A**) Far-UV CD spectra of CKm purified from young, middle-aged and aged mice. All CD experiments were conducted at 25 ˚C in 5 mM sodium phosphate buffer (pH 7.2) using hydroxyapatite purified CKm (>95% pure) at a protein concentration equal to 10 μM. (**B**) Limited chymotrypsin digestion of CKm purified from young, middle-aged and aged mice. Chymotrypsin was added to each sample and the reaction was quenched at 2, 5, 10, 20 and 40 minutes. Undigested CKm was used as the 0 minute time point. Time courses of proteolysis were constructed by resolving time points by SDS PAGE and staining gels with Coomassie blue. The abundance of undigested CKm was quantitated at each time point by performing densitometry on the 45 kDa band that corresponded to undigested CKm. Proteolysis experiments were repeated in triplicate and average density values were plotted versus reaction time. Linear regression analysis was used to plot best fit lines through the data and the slopes of these curves are given on the figure. Error bars represented standard error of mean calculated for each plotted value.

The secondary structure content of CKm purified by Blue Sepharose and hydroxyapatite fractionation from young, middle-aged, and aged mice was compared using far-UV CD spectrometry (Figure 4A; Table 2). CKm CD Spectra obtained for all three age groups show significant alpha helical character, however less ellipticity was observed in the middle-aged and aged samples relative to young CKm. The secondary structure composition of CKm purified from the differently aged mice was estimated by interpreting CD spectra using the SELCON3 program via the DICHROWEB server (www.cryst.bbk.ac.uk/cdweb/html/home.html) (Table 2); [20-22].  For example, sheet, turns and unordered protein segments. F_α__R_ (young) shows that the regular α-helix content is 25%.  Thus, in the aged sample there is a decrease in α-helical structure to 0.193 (19.3%) as indicated by F_α__R_ (aged).  Similarly the F_β__R_ and F_β__D_ which depict the percent regular β-sheet indicates, as expected, an age-associated increase in the distorted β-sheet.  Furthermore, there is an increase in unordered structure at the expense of the α-helix as indicated by the change in F_u_ from 0.276 (young) to 0.298) (aged).  Relative to the young CKm sample, the secondary structural composition of CKm from middle-aged and aged mice was characterized by decreased alpha helical content with concomitant increases in beta pleated sheet, turns and unordered protein segments.

**Table 2.** Predicted secondary structure content for CKm purified from differently agedmice.

	F_α__R_	F_αD_	F_α__Total_	F_β__R_	F_β__D_	F_β__Total_	F_T_	F_U_	Total	NRMSD
Young	0.250	0.159	0.409	0.090	0.068	0.158	0.162	0.274	1.002	0.274
Middle-Aged	0.236	0.158	0.394	0.096	0.071	0.167	0.172	0.274	1.011	0.308
Aged	0.193	0.144	0.337	0.095	0.073	0.168	0.187	0.298	0.990	0.285

### CKm from middle-aged and aged mice is charac-terized by increased susceptibility to proteolysis

The stability of CKm purified from differently aged mouse quadriceps was analyzed by limited chymotrypsin digestion and SDS PAGE (Figure 4B).  CKm purified from middle-aged and aged mice is digested approximately 3.5 times faster than CKm purified from young mice.  The rates of proteolysis are given on Figure 4B.  These results are consistent with several studies that have documented the proteolytic resistance of CKm [23]. Furthermore, Zhao et al. [7] showed that structural alteration of oxidized CK (O-CK) renders the enzyme more susceptible to proteolysis by both trypsin and proteinase K.   Regardless of age group, no proteolytic fragments were observed during the limited chymotrypsin digestion whereas the amount of full length protein decreases throughout the time course and the rates of proteolysis vary among age groups. Taken with the results from our CD experiments (Figure 4A) these data suggest that CKm purified from middle-aged and aged mice is less resistant to proteolysis suggesting that it is structurally less stable than CKm purified from young mice.

### Two proteins with molecular weights of 130 kDa and 88 kDa are immunoreactive with an anti-CKm antibody

**Figure 5.**
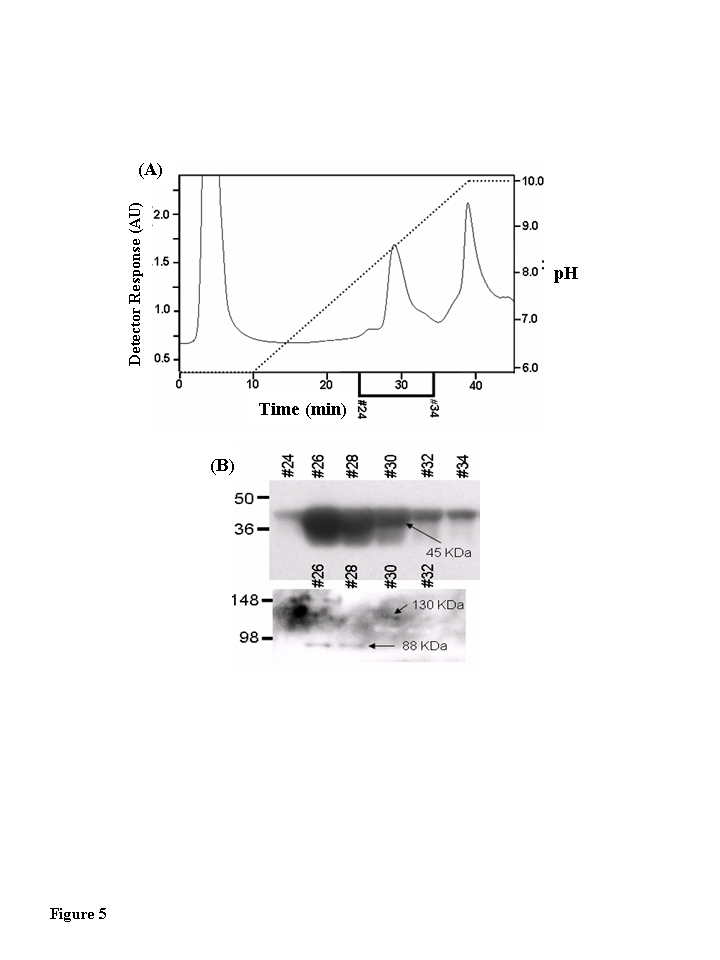
Muscle creatine kinase exists as 130 kDa and 88 kDa species *in vivo*. Blue Sepharose affinity column fractions of cytosolic protein from quadriceps of young, middle-aged, and aged mice that contained CKm were identified by Western blotting.  The samples were further fractionated by hydroxyapatite (HA) chromatography.  (**A**) HA fractionation of Blue Sepharose cytosolic quadriceps fractions from Blue Sepharose fractionation.  After application of samples to the HA columns, the loaded columns were washed with 10 ml of 50 mM sodium phosphate pH 5.8 and developed with a 30 ml  linear pH gradient (pH 5.8 to pH 10.0). Flow rate equaled 1 ml/min throughout purification and fractions were collected at a rate of one fraction per minute. The chromatograph in this figure represents fractionation of the middle-aged protein sample.  Fractionation of the young and aged samples yielded similar chromatographs. (**B)** Even numbered HA fractions (26-34, 20 μl) were resolved under denaturing conditions by SDS PAGE and transferred to a PVDF membrane. Blots were probed with an antibody specific for CKm.  A short exposure (15 second expo-sure, top blot) reveals that CKm (45 kDa) is abundant in fractions 26-34. A longer exposure (5 minutes, bottom blot) reveals additional CKm immunoreactive bands with higher molecular weights. A species with an apparent molecular weight of 88 kDa is observed in fractions 26 and 28, and a protein with an apparent molecular weight of 130 kDa is observed in fractions 30 and 32. The same high molecular weight CKm proteins were also observed after Blue Sepharose fractionation of young and aged mouse quadriceps samples.

Fractions 3-6 of the Blue Sepharose affinity column-linear pH gradient, shown by immunoblot to contain the unmodified CKm were fractionated by HA column chro- matography (Figure 5A).  These HA fractionations from all three age groups were analyzed for 3-NT modified proteins by resolving even numbered chromatography fractions on large format (26 wells) SDS polyacrylamide gels, transferring the resolved proteins to PVDF membranes, and probing the membranes with a monoclonal anti-3-nitrotyrosine antibody. Though 3-NT modified proteins were detected, none of the bands had molecular weights consistent with creatine kinase (data not shown). The anti-NT blots were stripped and reprobed with an anti-CKm antibody. Short exposure of the blots allowed CKm to be visualized within HA chromatography fractions 24-34 (Figure 5B, upper blot). To allow detection of less abundant CKm species the blots were exposed for 5 minutes. At these longer exposure times two protein bands with apparent molecular weights of 88 and 130 kDa were detected in HA fractions 26-32 (Figure 5B, lower blot). Throughout the rest of this manuscript, these proteins are referred to as CKm 88 and CKm 130, respectively.

### CKm 130 is 3-NT modified in an age-dependent manner

**Figure 6.**
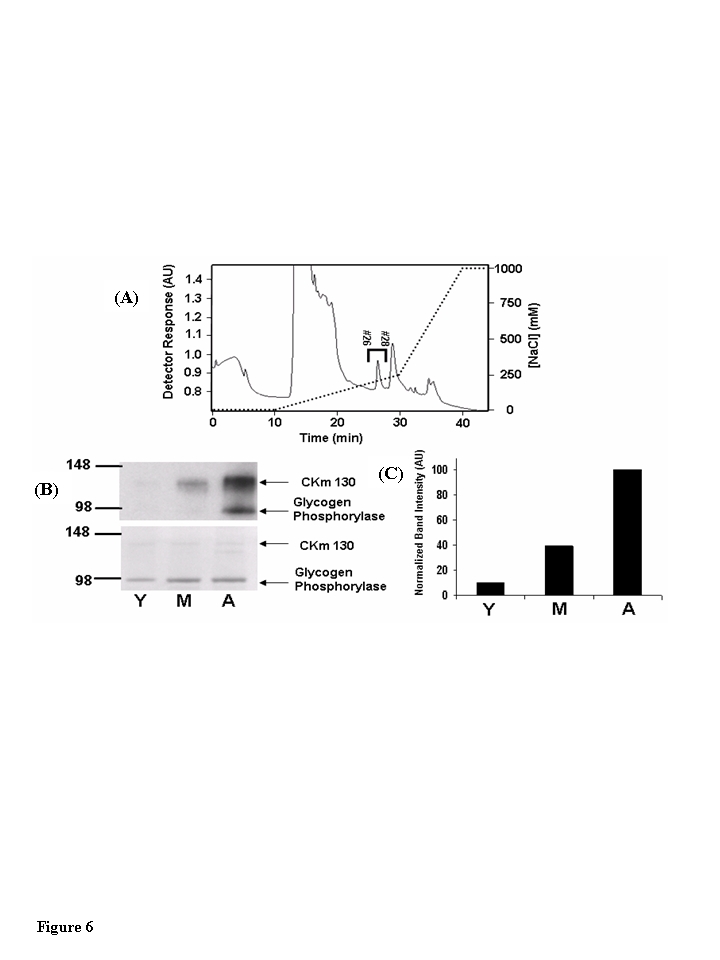
The 130 kDa CKm species is 3-nitrotyrosine modified in an age-dependent fashion. (**A**) Blue Sepharose protein fractions #26-#32 (see Figure 5) that contained anti-CKm immunoreactive proteins with apparent masses of 88 (CKm 88) and 130 (CKm 130) kDa were pooled from young, middle-aged and aged mouse samples and individually loaded onto a 1 ml mono-Q-Sepharose column (Biorad Laboratories). After application, samples were washed with 10 ml of 25 mM Tris pH 8.0, developed with a shallow 20 ml  linear NaCl gradient (0-250 mM NaCl in 25 mM Tris pH 8.0) followed by a steep 10 ml NaCl gradient (250 mM - 1M NaCl in 25 mM Tris pH 8.0). Flow rate equaled 1 ml/min throughout purification and fractions were collected at a rate of one fraction per minute. The above chromatograph was obtained by fractionation of the middle-aged protein sample, fractionation of the young and aged samples yielded similar chromatographs. Fractions 26-28 (indicated on the figure) contained CKm 130. These fractions were pooled and analyzed for 3-nitrotyrosine modification.  (**B**) Pooled protein (0.5 μg) from young (Y), middle-aged (M), and aged (A) Q-Sepharose fractionations were resolved by SDS PAGE and transferred to a PVDF membrane. Blots probed with an anti-3NT antibody [top blot, panel (B)] reveal that CK 130 is 3-NT modified in an age dependent manner. The membrane was then stained with Coomassie Blue [bottom blot, panel (B)] to normalize protein loading. (**C**) Densitometry was used to compare the relative abundance of the 3-NT modified form of CK 130 between age groups.

Anti-3NT Western blots of Blue Sepharose chromatography fractions revealed several 3-NT modified proteins with molecular weights ranging from 85-150 kDa (data not shown). To examine the possibility that CKm 88 and CKm 130 kDa are present in these high molecular weight 3-NT modified proteins, Blue Sepharose fractions 26-32 for each age group were pooled and further fractionated on a mono-Q-Sepharose anion exchange column (Figure 6A). Entire chromatography fractions from all three age groups were scanned for the 88 kDa and 130 kDa CKm proteins by resolving the even numbered fractions on large format (26 wells) SDS polyacrylamide gels, transferring the resolved proteins to PVDF membranes, and probing the membranes with an anti-CKm antibody. CKm with a molecular weight of 45 kDa (the predicted molecular weight of CKm) elutes from the column with a robust peak between 10 and 20 minutes (Figure 6A). The 130 kDa CKm species was observed in fractions 26 and 28; the 88 kDa CKm species was not detected.  Q-Sepharose fractions 26-28 were pooled from all three groups, concentrated by centrifugal filtration, and analyzed for 3- NT modification by SDS PAGE/Western blotting (Figure 6B, upper blot). Two proteins with apparent molecular weights of 130 and 100 KDa are 3-NT modified in an age-dependent manner (Figure 6B). In a parallel experiment, the bands that corresponded to the modified proteins were excised from a Coomassie Blue stained SDS-PAGE acrylamide gel. MALDI-TOF/TOF mass spectrometry analysis identified the modified proteins as CKm and glycogen phosphorylase (Table 1). Densitometric analysis of 3-NT modified CKm 130 reveals a progressive increase in 3-NT modification with age (Figure 6C).

**Steady state kinetic analysis of CKm shows age-associated decreases in the kinetic parameter Vmax**

**Figure 7.**
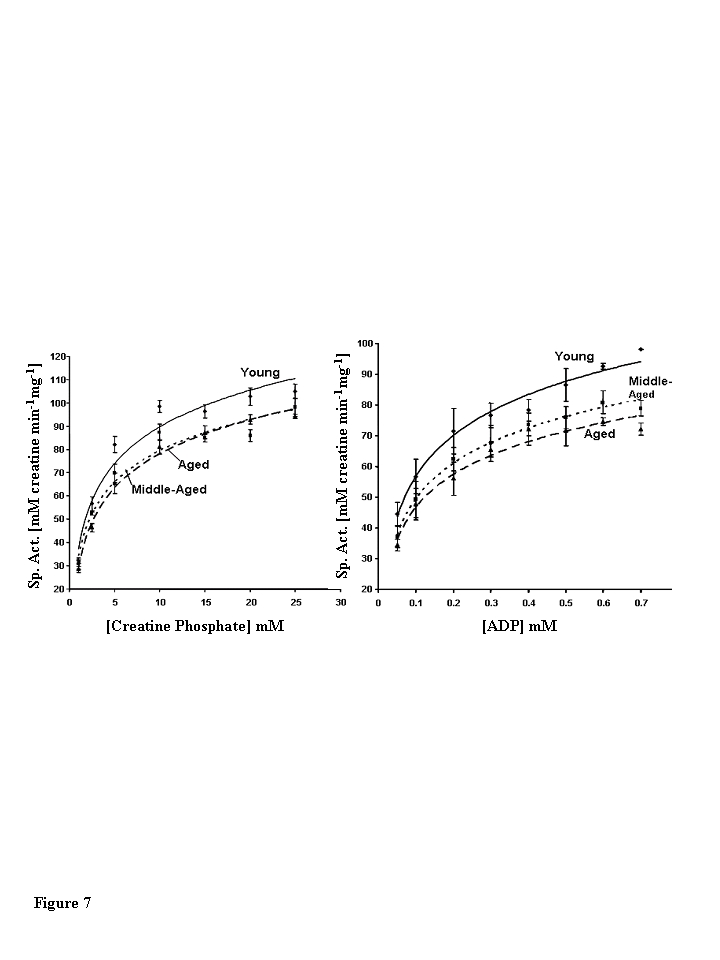
Steady state kinetic analysis of creatine phosphate. (**A**) Creatine phosphate and (**B**) ADP dependence of muscle creatine kinase purified from young (diamonds, solid line), middle-aged (squares, small dashed lined), and aged (triangles, large dashed line) mice.  The data presented in the figure are the average of four independent experiments for creatine phosphate and three independent experiments for ADP.  Nonlinear least squares regression analysis was used to plot best fit curves through the data. Error bars represent the standard error of mean calculated for each data point.

The substrate dependence of CKm activity was measured using a linked spectrophotometric assay system in the direction of creatine and ATP production [24,25]. Plots of initial reaction velocities vs. creatine phosphate and ADP concentrations exhibit hyperbolic Michaelis-Menton kinetics for CKm purified from all three age groups (Figure 7).  The parameters K_M_ and V_max_were extracted from Eadie-Hofstee plots (data not shown) of individual kinetic experiments (Table 3) [26,27].  Michaelis-Menton constant (K_M_) values measured for creatine phosphate and ADP agree well with previously published values and do not vary between age groups [25].  However, the V_max_decreases with age. The V_max _of middle-aged CKm is approximately 12.5% (13% decrease measured by creatine phosphate dependence and 12 % decrease measured by ADP dependence) less than the V_max _measured for the young enzyme (creatine phosphate dependence, p<0.005; ADP dependence, p<0.05). Interestingly, there were no statistically significant differences between kinetic parameters measured for middle-aged and aged CKm.

## Discussion

The decreased energy capacity of aging skeletal muscle, coupled with recent proteomic experiments, implicates diminished CKm function as a potential causative factor for age-related sarcopenia [8-12]. Our structural studies using CD spectrometry and limited chymotrypsin digestion are consistent with our hypothesis that the structural alteration due to oxidative modification may be a factor that affects CKm enzyme function. Although the CD spectra of young, middle-aged and aged CKm show significant characteristics of alpha helical structure in all three protein preparations [21,22,28], increases in beta-pleated sheet, turns, and unordered segments that occur with age suggest structural changes that are consistent with the observed decreases in enzyme activity. Furthermore, unfolding transitions associated with increases in beta-sheet and disordered segment content are associated with an increased tendency to aggregate, suggesting that middle-aged and aged CKm may be more prone to aggregation than young CKm [29,30]. Thus, we attribute the age-specific aggregation of CKm to the unfolding indicative of the increased beta sheet formation.  Also, the limited chymotrypsin digestion of middle-aged and aged CKm proceeded approximately 3.5 times faster than the digestion of the young. This is consistent with the observation that structural alteration of O-CK renders the enzyme more susceptible to proteolysis by both trypsin and proteinase K [7].  In light of the CD data, and the fact that the rate of limited chymotrypsin digestion increases 3.5-fold in modified CKm, we propose that the different rates of proteolysis result from age-related decreases in native state CKm stability.

The current study which directly examined the biochemical properties of CKm from mouse quadriceps revealed statistically significant decreases in V_max_, for middle-aged and aged CKm, relative to young CKm and no change in the kinetic parameter K_M_.  The biochemical consequences of the age-dependent decreases in V_max_, reflect slower rates of enzyme turnover in the middle-aged and aged muscle.  Interestingly, no statistically significant differences were observed between the middle-aged and aged V_max_ which suggests that even though the modifications continue to increase with age, those modifications that affect enzyme function may have occurred at middle age.

An underlying tenet of the Free Radical Theory of Aging is that age-related increases in ROS production and the concomitant increases in protein oxidation are gradual over a lifespan [31]. Moreover, proteins oxidized *in vitro* or *in vivo* often show decreased activity and stability, though there is significant variability in the extent of these changes [7,14-17].  Our data support the current interpretation of the Free Radical Theory of Aging as increased levels of nitration and carbonylation correlated with changes in function and structural features. However, the middle-aged and  aged CKm contained similar elevated levels of carbonyls, *i.e.,* approximately 2.5 times more than young CKm, data which support a model of muscle aging where the majority of this specific age-related modification un-expectedly occurs by middle-age. Given the complexity of *in vivo* oxidative stress and variability in the intrinsic ROS resistance of different proteins, it is not surprising that some proteins may show differential levels of sensitivity to oxidative modification at middle age.

Though the observed changes in structure and function correlated with nitration and carbonyl levels, other covalent oxidative modifications that result in altered structure and function support our studies.  Interestingly, the initial loss of GAPDH activity due to oxidative nitrative stress has been shown to occur prior to the detection of its nitration [32,33].  It has been proposed that this maybe due to oxidation of cysteines of the GAPDH active site.  It is possible therefore, that cysteine oxidation may be a factor in the loss of CKm activity in middle aged muscle. Oxidation of Cys^74^ and Cys^146^ which forms the intrachain disulfide bond in oxidized CKm (O-CKm) causes dramatic structural changes that affect the dimerization interphase and results in decreased catalytic activity, structural instability, failure to interact with the M-line protein myomesin, and ubiquitination [7].  The latter targets O-CKm for ATP-ubiquitin proteo-some degradation and suggests that the generation of O-CKm is a negative regulatory mechanism that may play a role in CKm turnover.  Furthermore, Cys^283 ^ of the active site is essential for catalysis and is a plausible site of oxidative modification during aging [34].  In the O-CKm model, the orientation of Cys^283^ is altered which may be an additional cause for decreased catalytic activity. These PTMs, strongly suggest that the structural alterations caused by nitration and/or carbonylation that we have identified may be the cause for loss of function in the aged muscle.

**Table 3.** Creatine kinase kinetic parameters.

	_Creatine Phosphate Dependence_		_ADP Dependence_	
	K_M _(mM)	V_max _(sp. Activity)	K_M _(mM)	V_max _(sp. Activity)
CK_young_	2.6 +/- 0.1	117 +/- 1.6**	66.7 +/- 11	98 +/- 2.2 *
CK_middle_	2.3 +/- 0.2	102 +/- 2.9**	70.8 +/- 8.6	86 +/- 3.5 *
CK_aged_	2.8 +/- 0.2	103 +/- 2.7	70.7 +/- 8.2	81 +/- 3.7

We and others have shown that CKm is 3-NT modified within urea and detergent solubilized muscle extracts [10,11]. Our Blue Sepharose fractionation confirmed the presence of 3-NT modified form of CKm under native conditions, but interestingly it showed for the first time that this modification altered the chromatographic properties of nitrated CKm as indicated by the shift in its elution.  While protein nitration is well documented as a marker of oxidative stress it is also recognized that tyrosine nitration affects both structure and function of the modified protein. Nitrotyrosine shifts the pKa of the targeted region of the tyrosine ring structure by approximately 3 pH units [35], and introduces steric and electrostatic alterations in protein structure [36].  These altered characteristics may explain the shift in elution of nitrated CKm in the Blue Sepharose fractionation.  Furthermore, our results also indicate that the nitrated CKm fractions show very low levels of carbonylation suggesting that the chromatographic shift may be due to structural changes caused by the nitration.  Formation of the age-specific, CKm immunoreactive 130 kDa protein suggests that oxidative modification may cause structural changes that lead to aggregation. The observed molecular weight of the protein and the fact that mass spectrometry analysis did not produce significant search scores for other proteins, suggest that this is an SDS-stable, trimeric form of CKm. We also observed an 88 kDa protein by Western blot analysis, consistent with the formation of an SDS-stable dimeric form. Perhaps the most likely structural explanation for these species is a covalent cross-linking of two and three CKm subunits, respectively, although there are reports of ROS-induced noncovalent oligomers that are resistant to SDS denaturation [37]. To our knowledge this is the first report of these age- CKm 88 and CKm 130 species.

It is interesting that nitration of CKm 45 (monomer), CKm 88 (dimer) and CKm 130 (trimer) were observed to significantly increase with age. Based on these observations, we hypothesize that within the cell, the 3-NT modified CKm is affiliated with age-associated protein aggregation. This contention is supported by a report which described the use of a fluorescent probe (bis-ANS) to monitor protein conformation within muscle extracts [12]. The low bis-ANS fluorescence quantum yields observed within aged skeletal muscle samples are consistent with increased incidences of CKm protein aggregation with age. It is likely that this is not a unique observation for CKm but is a general consequence of age-related protein oxidation and nitration [38-40]. Based on those observations we hypothesize that (a) modified proteins may accumulate in aged tissue because of this aggregation; (b) aggregation due to oxidative damage per se is not catastrophic but may account for the decline in tissue function; (c) these low levels of aggregated proteins may act as "seeds" and increase aggregation in catastrophic misfolded protein syndromes; (d) these low levels of aggregated proteins may elicit a misfolded protein stress response that would account for the stabilization of the age-associated increase in state-of-chronic stress [41,42].

While our study has examined some of the structural and functional consequences of oxidative modification of CK, there are other potential effects of age-related oxidative modification.  One area which remains to be examined is the possibility that carbonylation and/or nitration may alter protein-protein interactions of CK.  It has been reported that a subpopulation consisting of approximately 5-10% of the muscle isoform of CK associates with the M line area of the sarcomere [43].

This positions muscle CK in proximity to the myosin ATPase, potentially to provide a ready pool of ATP to power muscle contraction [6].  Mutagenesis experi-ments have shown that two pairs of lysine residues in the amino terminal of CK are critical for the association of CK with the M-line area.  Endogenous brain-type CK expressed in skeletal muscle does not associate with the M line; however, insertion of the lysine pairs from CKm into the amino terminal of brain-type CK confers the ability to associate with the M line [3].

Lysine is a target of carbonylation.  We have shown an age-dependent increase in the degree of carbonylation of CKm.  It is possible that the amino terminal lysines which are critical for the association of CKm with the M line may be targets of age-related carbonylation, which in turn could affect the ability of CK to bind to the M line.  This potential loss of protein-protein interaction, in addition to the decreased enzymatic activity which we have shown in this paper, could have the effect of decreasing the local concentration of ATP available to the sarcomere in aged skeletal muscle, leading to decreased muscle function.  Similarly, since Tyr^14^ and Tyr^20^ are nitrated in aged rat skeletal muscle [11] this modification of the CKm amino terminal domain also alters the ability to bind to the M line thereby decreasing local concentration of ATP available to the sarcomeres and decrease function of aged muscle [4,5,7].  Alternatively, the recent observation that O-CKm causes a major change in orientation of Lys^25^ and Lys^116^ may also account for the loss the ability of O-CKm to bind to the M-band [7].

Proteomics experiments have identified a growing list of proteins that are differentially modified by carbonylation or nitration during aging [10,11,43-46]. These approaches provide valuable insight in that they identify specific proteins whose functions may be altered. In this study we attempt to further understand the biochemical consequences and biological significance of the age-related oxidation and nitration of CKm.  We observed higher levels of carbonylation within middle-aged and aged CKm samples, relative to young CKm which is affiliated with a significant reduction in function by middle-age.  Unlike carbonylation of the protein, 3-NT modification of the CKm appears to increase throughout lifespan and we hypothesize that this modification is affiliated with altered protein function. It is possible that the tyrosine nitration of CKm only occurs on molecules that are already substantially oxidized. Kanski et al [11] have mapped the sites of modification to Tyr^14^ and Tyr^20^ in rat skeletal muscle CKm. Inspection of the solvent accessibility of these residues within the rabbit enzyme (pdb identifier: 2CRK) using the GETAREA server (www.scsb.utmb.edu/cgi-bin/get_a_form.tcl) revealed that though Tyr^14^is on the outside of the protein, Tyr^20^ is partially buried having a solvent accessible surface area of only 21.4 % [20,43]. Perhaps, the age-related oxidation of CKm induces partial unfolding, where higher levels of reactive nitrogen species within aged muscle leads to tyrosine nitration which induces an unfolded conformation that is no longer soluble.

Our studies suggest that the structural changes of the nitrated Ckm in aged skeletal muscle may play an important role in its aggregation.  However, since aggregated proteins are normally cleared by autophagy, our studies also suggest that CKm accumulation may be due to an attenuated autophagy in aged skeletal muscle. Furthermore, since the accumulation of misfolded and aggregated proteins is known to play a major role in neurodegeneraction [51], we propose that aggregation and loss of function of CKm in aged skeletal muscle may play a role in age-associated skeletal muscle frailty such as sarcopenia and musculoskeletal disorders.

Overall, the results from this study suggest that reduction in CKm activity could contribute to the decreased oxidative capacity of aged skeletal muscle. Additionally, another enzyme involved in energy availability within muscle, glycogen phosphorylase, is 3-NT modified in an age-dependent manner. Sharov et al. [48] have reported a decrease in glycogen phosphorylase isolated from aged rat muscle that correlated with an age-dependent increase in 3-NT modification. The study did not examine the properties of the enzyme from a middle-aged sample but our results show significant 3-NT modification of glycogen phosphorylase (GP) obtained from middle-aged mouse quadriceps. Based on these data, we predict that GP activity would be significantly decreased by middle-age as well, though the level of reduction in activity would likely be less than the approximate 30% decrease reported for aged GP.  Given the importance of CKm and GP, it seems increasingly likely that oxidative and nitrative modification leads to reductions in function of these two key enzymes, decreasing the availability of essential energy metabolites which directly contribute to the aged skeletal muscle phenotype.

## Methods

Materials . Tris-HCl, ADP, alpha D-glucose, NADP, creatine phosphate, dithiothreitol, hexokinase, and glucose-6-phosphate dehydrogenase were purchased from Sigma-Aldrich. Imperial Coomassie Blue protein stain was obtained from Pierce. All other reagents were from standard suppliers and were at least reagent grade.  All solutions were prepared in Milli-Q (Millipore) doubly deionized water.

Mice. Young (3-6 months), middle-aged (12-14 months) and aged (20-24 months) male C57BL/6 mice from the National Institute on Aging colonies (Bethesda, MD) were obtained through Charles River Laboratories (Wilmington, MA). Mice were maintained with a 12 hour light/dark cycle and fed *ad libitum* on a standard chow diet for at least one week before use. Mice were sacrificed by cervical dislocation. All mice used in this study were free of tumors or any other gross pathological conditions. The quadriceps muscles were harvested and snap frozen in liquid nitrogen until analysis.

Total quadriceps tissue extracts. Whole protein extracts were prepared from the quadriceps muscles of six young (3-6 months), six middle-aged (12-14 months), and five aged (20-24 months) C57BL/6 male mice. Whole muscle extracts were prepared from individual samples by grinding the quadriceps with an abrasive resin (PlusOne Sample Grinding Kit, Amersham Biosciences) in urea/CHAPS buffer (8M urea, 4% CHAPS) following the manufacturer's recommendations. After homogenization, insoluble material was cleared with a 30 minute centrifugation (8,000 x g) and the supernatants were retained for immunoblot analysis. Protein was quantified with the Bradford protein assay (Biorad), using BSA as a standard.

Preparation of soluble protein extracts from quadriceps. Quadriceps muscles obtained from five young, five middle-aged and five aged C57BL/6 male mice were pooled and homogenized with a whirling Polytron blender in a nondenaturing buffer (50 mM sodium phosphate (pH 5.8), 1mM DTT, 0.4 mM EDTA and 1mM PMSF). Insoluble material was cleared with a 30 minute centrifugation (8,000 x g) and the supernatant was retained for protein purification.

Purification of creatine kinase from young, middle-aged and aged mouse quadriceps. All column chromatography steps were performed on a dual pump HPLC system (ESA Biosciences) equipped with a UV-Vis detector (UV-Vis Model 528, ESA Biosciences) and a Gilson FC 204 fraction collector. The purification of CKm was based on previously published methods with slight modifications [49]. Soluble protein from young, middle-aged, and aged mice quadriceps were applied to a 5 ml Blue Sepharose affinity column (Amersham Biosciences, HiTrap Blue HP). The resin was washed with 25 ml of mobile phase (50 mM sodium phosphate, pH 5.8) and protein eluted with 50 mM sodium phosphate (pH 8.5). Fractions containing CKm, as determined by Western blot, were pooled, diluted 1 to 10 in 50 mM sodium phosphate (pH 5.8) and reapplied to the Blue Sepharose column. The column was washed and CKm was eluted with a 50 ml linear pH gradient (pH 5.8 to pH 10.0). Flow rate throughout Blue Sepharose chromatography equaled 1 ml/min and fractions were collected at a rate of one fraction every minute. Peak fractions, containing CKm, were greater than 85% pure as determined by densitometry and were pooled and saved for kinetic and protein immunoblot analysis that compared levels of carbonylation and 3-NT modification.  Side fractions, approximately 50% pure, were pooled and applied to a 2 ml Bio-Scale ceramic hydroxyapatite (HAP) column (Bio-Rad Laboratories).  After the column was washed with 10 ml of low phosphate buffer (5mM sodium phosphate, pH 7.4), protein was eluted with a linear sodium phosphate (pH 7.4) gradient (5mM - 150 mM). Throughout HAP chromatography, the flow rate equaled 0.5 ml/min and fractions were collected at a rate of one fraction every minute. Peak fractions, used in circular dichroism and limited proteolysis studies, were greater than 95% pure as determined by densitometry following SDS PAGE. Concentrations of purified CKm samples were determined by optical density measurements at 280 nm using an extinction coefficient of 0.876 ml•mg^-1^•cm^-1^ [50].

Enrichment of high molecular weight CKm protein species. Soluble quadriceps protein from all three age groups was fractionated on a 5 ml Blue Sepharose affinity column (Amersham Biosciences, HiTrap Blue HP). After sample loading, the column was washed with 10 ml of 50 mM sodium phosphate pH 5.8 and developed with a 30 ml linear pH gradient (pH 5.8 to pH 10.0). Flow rate equaled 1 ml/min throughout purification and fractions were collected at a rate of one fraction per minute. Fractions containing anti-CKm immuno-reactive proteins with apparent masses of 88 and 130 kDa were pooled from young, middle-aged and aged mouse samples and individually loaded onto a 1 ml mono-Q-Sepharose column (Bio-Rad Laboratories). After application, the column was washed with 10 ml of 25 mM Tris (pH 8.0), developed with a shallow 20 ml  linear NaCl gradient (0-250 mM NaCl in 25 mM Tris pH 8.0), followed by a steep 10 ml NaCl gradient (250mM - 1M NaCl in 25 mM Tris pH 8.0). The flow rate equaled 1 ml/min throughout purification and fractions were collected at a rate of one fraction per minute.

Creatine kinase activity assay. Creatine kinase activity was assayed in the direction of creatine and ATP production using a linked spectrophotometric assay [24,25]. Final concentrations for the assay were: alpha D-glucose (15 mM), ADP (50-700 μM), MgCl_2_ (9.0 mM), NADP (1.3 mM), creatine phosphate (1-25 mM), DTT (9.0 mM), Hexokinase (2.5 mU/ml), and Glucose-6-phosphate dehydrogenase (2.5 mU/ml). All reagents were prepared in 50 mM Tris-HCl, pH 7.4. All CKm activity measurements were made at 25˚C on a Beckman Coulter DU530 spectrophotometer.

Enzyme kinetics. CKm activity, as a function of substrate concentrations, was measured by varying creatine phosphate between 1 mM and 25 mM and ADP between 50 μM and 700 μM. Initial reaction velocities were determined by measuring the initial change in absorbance at 340 nm and converting the data to units of specific activity (μmols creatine min^-1^mg^-1^) using an extinction coefficient of 6220 M^-1^cm^-1^. Four and three independent experiments were performed, for substrate and co-factor, respectively and the kinetic parameters, K_M_ and V_max_, were calculated from Eadie-Hofstee plots obtained from individual kinetic experiments [26,31]. Standard errors of mean were also calculated for each parameter.

SDS PAGE and western blot analysis. Proteins were resolved on denaturing 4-20% gradient gels (PAGE Gold precast gels, Cambrex Corporation) and transferred to Immobolin PVDF membranes (Millipore) at 50 V for two hours. Membranes were blocked with 5% nonfat milk in TBS-T and probed with primary antibodies. A monoclonal anti-3-nitrotyrosine (Upstate Biotechnology) antibody and a polyclonal anti-creatine kinase M (Santa Cruz Biotechnology) antibody were used to detect 3-nitrotyrosine modified proteins and the muscle isotype of creatine kinase, respectively. Blots were visualized with appropriate horse radish peroxidase conjugated secondary antibodies (Alpha Diagnostic) used in conjunction with a chemi-luminescent substrate (Immobilon Western Blot reagent, Millipore). Kodak BioMax MR film was used to visualize specific antibody binding. Exposed films of immunoblots were digitized using a MultImage imaging system (Alpha Innotech Corporation) and quantified by densitometry using AlphaEase software (Alpha Innotech Corporation). Statistical analysis was performed by comparing normalized immunoblot band densities using the 2-tailed t-test. P-values less than 0.05 were considered statistically significant.

Detection of oxidized creatine kinase. The relative abundance of oxidative modifications (carbonyls) in young, middle-aged and aged CKm preparations were determined using the Oxyblot kit  (Intergen Company; 18, 19). Carbonyls within protein samples were detected following the manufacturer's recommendations with slight modification. Briefly, 1 μg of CKm purified from each age group (Blue Sepharose pools, > 85% pure) was derivatized with 2,4-dinitrophenylhydrazine for exactly 10 minutes. After derivatization reactions were quenched, samples resolved by SDS-PAGE, and transferred to a PVDF membrane.  Blots were developed using a primary antibody that is specific for the 2,4-dinitrophenylhydrazone (DNP) moiety and blots visualized with an appropriate HRP-conjugated secondary antibody. Blots were quantified by densitometry and the Coomassie Blue stained membrane was used to normalize for sample loading variation.

Circular dichroism. CKm samples (HAP purified, > 95 % pure) from all three age groups were dialyzed versus 5 mM sodium phosphate buffer (pH 7.2) and each sample diluted to a concentration of 10 μM in preparation for circular dichroism (CD) analysis. CD wavelength scans were made at 25˚ C in a 0.1 cm path length cuvette in the far-UV region (195 - 255 nm). CD measurements were made on an Aviv (Aviv Instruments) Model 215 CD Spectrometer. CD spectra were acquired by averaging three scans and subtracting buffer absorbance. The secondary structure composition within each sample was estimated using the program SELCON3 as accessed via the DICHROWEB server at (http://www.cryst.bbk.ac.uk/cdweb/html/home.html) [21,22,28].

Limited proteolysis. Limited proteolytic digestions of CKm were performed on hydroxyapatite purified samples  (> 95% pure) by adding chymotrypsin (Worthington Chemicals) to a concentration of 1/20 (w/w) and allowing the reaction to proceed at room temperature for 2, 5, 10, 20, and 40 minutes. At the indicated time points, a 1 μg aliquot was removed and diluted into 5X SDS sample loading buffer. Proteolysis was quenched by boiling the samples for 5 minutes. Undigested CKm was used as the "0 minute" time point for all three age groups. Time courses were then resolved by SDS PAGE and proteins visualized by Coomassie Blue staining. All proteolytic time courses were repeated three times. Densitometric calculations were used to quantify the relative abundance of undigested CKm throughout the time courses.

Protein identification. Protein bands were excised from Coomassie Blue stained gels and prepared for Matrix-Assisted Laser Desorption Ionization Time-of-Flight mass spectrometry (MALDI-TOF) analyses.  Gel pieces were incubated with trypsin (20 μg/ml in 25 mM ammonium bicarbonate, pH 8.0; Promega Corp.) at 37˚C for 6 hours.  The digested sample (1 μL) was deposited onto the MALDI plate and allowed to dry.  Matrix (1 μL; alpha-cyano-4-hydroxycinnamic acid; Aldrich Chemical Company) was then applied on the sample spot and allowed to dry.  MALDI-TOF/TOF MS was performed using an Applied Biosystems model 4700 Proteomics Analyzer for peptide mass fingerprinting and MS/MS analysis.  Following MALDI MS analysis, MALDI MS/MS was performed on several ions from each sample spot.  Applied Biosystems GPS software was used in conjunction with MASCOT to search the NCBI database for protein identification.  Protein match probabilities were deter-mined using expectation values and MASCOT protein scores.

Calculation of solvent accessible surface area of CKm residues. The solvent accessible surface area (SASA) of each amino acid of CKm was calculated by submitting the atomic coordinates of the rabbit CKm crystal structure (pdb identifier: 2CRK) to the GETAREA server (http://www.scsb.utmb.edu/cgi-bin/get_a_form.tcl) [20,43]. Default parameters were used for calculating the percent solvent accessibility of each residue.

## References

[1] McLeish MJ, Kenyon GL (2005). Relating structure to mechanism in creatine kinase. Crit Rev Biochem Mol Biol.

[2] Eppenberger HM, Dawson DM, Kaplan NO (1967). The comparative enzymology of creatine kinase. J Biol Chem.

[3] Hornemann T, Stolz M, Wallimann T (2000). Isoenzyme-specific interaction of muscle-type creatine kinase with the sarcomeric M-line is mediated by NH(2)-terminal lysine charge-clamps. J Cell Biol.

[4] Hornemann T, Kempa S, Himmel M, Hayess K, Furst DO, Wallimann T (2003). Muscle-type creatine kinase interacts with central domains of the M-band proteins myomein and M-protein. J Mol Biol.

[5] Wallimann T, Schlosser T, Eppenberger HM (1984). Function of M-line-bound creatine kinase as intramyofibrillar ATP regenenera-tor at the receiving end of the phosphorylcreatine shuttle in muscle. J Biol Chem.

[6] Ventura-Clapier R, Veksler V, Hoerter JA (1994). Myofibrillar creatine kinase and cardiac contraction. Mol Cell Biochem.

[7] Zhao T-J, Yan Y-B, Liu Y, Zhou H-M (2007). The generation of oxidized form of creatine kinase is a negative regulation on muscle creatine kinase. J Biol Chem.

[8] Taylor DJ, Kemp GJ, Thompson CH, Radda GK (1997). Ageing: Effects on oxidative function of skeletal muscle in vivo. Mol Cell Biochem.

[9] Pastoris O, Boschi F, Verri M, Baiardi P, Felzani G, Vecchiet J, Dossena M, Catapano M (2000). The effects of aging on enzyme activities and metabolite concentrations in skeletal muscle from sedentary male and female subjects. Exp Gerontol.

[10] Kanski J, Behring A, Pelling J, Schoneich C (2005). Proteomic identification of 3-nitrotyrosine-containing rat cardiac proteins: effects of biological aging. Am J Physiol Heart Circ Physiol.

[11] Kanski J, Hong SJ, Schoneich C (2005). Proteomic analysis of protein nitration in aging skeletal muscle and identification of nitrotyrosine-containing sequences in vivo by nanoelectrospray ionization tandem mass spectrometry. J Biol Chem.

[12] Pierce A, deWaal E, VanRemmen H, Richardson A, Chaudhuri A (2006). A novel approach for screening the proteome for changes in protein conformation. Biochem.

[13] Smith CD, Carney JM, Starkreed PE, Oliver CN, Stadtman ER, Floyd RA, Markesbery WR (1991). Excess brain protein oxidation and enzyme dysfunction in normal aging and in Alzheimer-Disease. Proc Natl Acad Sci.

[14] Rothstein M, Roy AK, Chatterjee B (1984). Changes in enzymatic proteins during aging. Molecular Basis of Aging.

[15] Oliver CN, Ahn BW, Moerman EJ, Goldstein S, Stadtman ER (1987). Age-Related-Changes in Oxidized Proteins. J Biol Chem.

[16] Zhou JQ, Gafini A (1991). Exposure of rat muscle phosphoglycerate kinase to a nonenzymatic Mfo system generates the old form of the enzyme. J Gerontol.

[17] Szweda LI, Stadtman ER (1992). Iron-Catalyzed Oxidative Modifica-tion of Glucose-6 Phosphate Dehydrogenase from Leuconostoc-Mesenteroides - Structual and functional Changes. J Biol Chem.

[18] Levine RL, Wehr N, Williams JA, Stadtman ER Shacter E (2000). Determination of carbonyl groups in oxidized proteins. Methods Mol Biol.

[19] Levine RL, Stadtman ER (2001). Oxidative modification of proteins during aging. Exp Gerontol.

[20] Fraczkiewicz R, Braun W (1998). Exact and efficient analytical calculation of the accessible surface areas and their gradients for macromolecules. J Comput Chem.

[21] Lobley A, Whitmore L, Wallace BA (2002). DICHROWEB: an interactive website for the analysis of protein secondary structure from circular dichroism spectra. Bioinformatics.

[22] Whitmore L, Wallace BA (2004). DICHROWEB, an online server for protein secondary structure analyses from circular dichroism spectroscopic data. Nucleic Acids Res.

[23] Wyss M, James P, Schlegel J, Wallimann T (1993). Limited proteolysis of creatine-kinase - implications for 3-dimensional structure and for conformational substrates. Biochem.

[24] Rosalki SB (1967). An improved procedure for creatine phosphokinase determination. J Lab Clin Med.

[25] Basson CT, Grace AM, Roberts R (1985). Enzyme-Kinetics of a highly purified mitochondrial creatine-kinase in comparison with cytosolic forms. Mol Cell Biochem.

[26] Eadie GS (1942). The inhibition of cholinesterase by physostigmine and prostigmine. J Biol Chem.

[27] Hofstee BJH (1959). Non-inverted versus inverted plots in enzyme kinetics. Nature.

[28] Sreerama N, Venyaminov SY, Woody RW (1999). Estimation of the number of alpha-helical and beta-strand segments in proteins using circular dichroism spectroscopy. Protein Science.

[29] Dong AC, Prestrelski SJ, Allison SD, Carpenter JF (1995). Infrared Spectroscopic Studies of Lyophilization-Induced and Temperature-Induced Protein Aggregation. J Pharm Sci.

[30] Chi EY, Krishnan S, Randolph TW, Carpenter JF (2003). Physical stability of proteins in aqueous solution: Mechanism and driving forces in nonnative protein aggregation. Pharm Res.

[31] Stadtman ER, Levine RL (2000). Protein oxidation. Ann N Y Acad Sci.

[32] Buchczyk DP, Grune T, Sies H, Klotz LO (2003). Modifications of glyceraldehyde-3-phosphate dehydrogenase induced by increasing concentrations of peroxynitrite: early recognition by 20S proteasome. Biol Chem.

[33] Schroeder P, Klotz LO, Buchczyk DP, Sadik CD, Schewe T, Sies H (2001). Epicatechin selectively prevents nitration but not oxidation reactions of peroxynitrite. Biochem Biophys Res Comm.

[34] Buechter DD, Medzihradszky KF, Burlingame AL, Kenyon GL (1992). The active-site of creatine kinase-affinity labeling of cysteine-282 with N-(2,3-epoxypropyl)-N-amidinoglycine. J Biol Chem.

[35] Souza JM, Peluffo G, Radi R (2008). Protein tyrosine nitration-functional alteration or just a biomarker. Free Rad Biol Med.

[36] Savvides SN, Scheiwein M, Bohme CC, Arteel GE, Karplus PA, Becker K, Schirmer RH (2002). Crystal structure of the antioxidant enzyme glutathione reductase inactivated by peroxynitrite. J Biol Chem.

[37] Chapman ALP, Winterbourn CC, Brennan SO, Jordan TW, Kettle AJ (2003). Characterization of non-covalent oligomers of proteins treated with hypochlorous acid. Biochem J.

[38] Hyun DH, Gray DA, Halliwell B, Jenner P (2004). Interference with ubiquitination causes oxidative damage and increased protein nitration: implications for neurodegenerative diseases. J Neurochem.

[39] Agbas A, Zaidi A, Michaelis EK (2005). Decreased activity and increased aggregation of brain calcineurin during aging. Brain Research.

[40] Hawkins CL, Davies MJ (2005). The role of reactive N-bromo species and radical intermediates in hypobromous acid-induced protein oxidation. Free Rad Biol Med.

[41] Hsieh C-C, Papaconstantinou J (2002). The effect of aging on p38 signaling pathway activity in the mouse liver and in response to ROS generated by 3-nitropropionic acid. Mech Aging Dev.

[42] Hsieh C-C, Papaconstantinou J (2006). Thioredoxin-ASK1 complex levels link mitochondrial ROS activation for the p38 MAPK pathway in aged mouse livers. FASEB J.

[43] Turner DM, Walker JB (1987). Enhanced ability of skeletal muscle containing cyclocreatine phosphate to sustain ATP levels during ischemia following beta-adrenergic stimulation. J Biol Chem.

[44] Rabek JP, Boylston WH, Papaconstantinou J (2003). Carbonylation of ER chaperone proteins in aged mouse liver. Biochem Biophys Res Comm.

[45] Reverter-Branchat G, Cabiscol E, Tamarit J, Ros J (2004). Oxidative damage to specific proteins in replicative and chronological-aged Saccharomyces cervisiae - Common targets and prevention by calorie restriction. J Biol Chem.

[46] Sharma R, Nakamura A, Takahashi R, Nakamoto H, Goto S (2006). Carbonyl modification in rat liver histones: decrease with age and increase by dietary restriction. Free Rad Biol Med.

[47] Rao JK, Bujacz G, Wlodawer A (1998). Crystal structure of rabbit muscle creatine kinase. Febs Lett.

[48] Sharov VS, Galeva NA, Kanski J, Williams TD, Schoneich C (2006). Age-associate tyrosine nitration of rat skeletal muscle glycogen phosphorylase b: characterization by HPLC-nanoelectrospray-tandem mass spectrometry. Exp Gerontol.

[49] Fisher SE, Whitt GS (1979). Purification of the creatine-kinase isozymes of the green sunfish (Lepomis-Cyanellus) with Blue Sepharose C1-6B. Anal Biochem.

[50] Kuby S, Noda L, Lardy H (1954). Adenosinetriphosphate-creatine transphosphorylase. I. Isolation of the crystalline enzyme from rabbit muscle. J Biol Chem.

[51] Komatsu M, Waguri S, Chiba T, Murata S, Iwata JI, Tanida I, Ueno T, Koike M, Uchiyama Y, Kominami E, Tanaka K (2006). Loss of autophagy in the central nervous system cuases neuro-degeneration in mice. Nature.

